# Is malaria over-diagnosed? A world malaria day 2017 experience by Excellence and Friends Management Care Centre (EFMC) and partners, Abuja Nigeria

**DOI:** 10.11604/pamj.2017.28.273.12732

**Published:** 2017-11-28

**Authors:** Obinna Ositadimma Oleribe, Princess Osita-Oleribe, Ekei Ekom, Oriaku Ofem, Chidi Igwesi, Obison Guy Chigozie, Munaonyeso Ekweghariri, Grace Iyalla, Simon David Taylor-Robinson

**Affiliations:** 1 Excellence and Friends Management Care Centre (EFMC), N° 8 Excellence and Friends Street, Dutse, Abuja Nigeria; 2 Centre for Family Health Initiative (CFHI), Plot 508 Excellence and Friends Road, Kubwa, Abuja Nigeria; 3 Royal College of Physicians of London, 11 St Andrews Place, Regent’s Park, London NW1 4LE; 4 Faculty of Medicine, Imperial College London, St Mary’s Hospital Campus, Norfolk Place, London W2 1PG, United Kingdom

**Keywords:** EFMC, Malaria, over-diagnosis, rapid diagnostic test, Nigeria

## Abstract

Malaria remains a major cause of mortality across the world, but particularly in sub-Saharan Africa. WHO-sponsored World Malaria Day activity has helped to improve education and has contributed to a reduction in mortality globally in the past decade. However, much needs to be done still in Africa. We report on a World Malaria Day scheme in three primary Healthcare Facilities in and around the Abuja Federal Capital Territory in Nigeria in 2017. Activity included educational talks to pregnant women and nursing mothers of young children, with malarial testing, distribution of free mosquito nets and also medical treatment if needed. We found a large clinical over-diagnosis of malaria with simple fevers of any cause being reported as malaria. None of these cases were found to be due to malaria on formal malarial testing. We conclude that efforts should continue into education and prevention of malaria with insecticide-impregnated mosquito nets a key factor. However, over-diagnosis of malaria and the use of unnecessary antimalarial treatment may lead to parasite resistance to antimalarial treatment, morbidity from drug side-effects and potential mortality from not receiving the right treatment for other febrile illnesses. We recommend that malarial testing, particularly with simple blood film microscopy is implemented more widely across Africa, as it is simple to perform and allows effective management plans to be drawn up for individual patients.

## Introduction

Malaria is a preventable and curable/treatable illness transmitted by infected female Anopheles mosquitoes. According to the World Health Organization (WHO), in 2015, 91 countries and areas had ongoing malaria transmission [1]. However, increased efforts are dramatically reducing the malaria burden in many places. Between 2010 and 2015, malaria incidence among populations at risk fell by 21% globally [1]. In that same period, malaria mortality rates among populations at risk fell by 29% globally among all age groups and by 35% among children under 5. However, the WHO African Region carries a disproportionately high share of the global malaria burden and was home to 90% of malaria cases and 92% of malaria deaths in 2015 [1]. In sub-Saharan Africa, the high malaria burden is coupled with high rates of poverty and very low access to adequate health care. Efforts aimed at preventing the spread of malaria are very important to reduce the malaria burden. This informed the theme for 2017 World Malaria Day (WMD) with “a push for prevention” given that malarial drug resistance is increasing, the prevention of malaria is pertinent to reducing deaths and protecting vulnerable people, such as nursing mothers and young children. In aligning with this year's prevention drive, Excellence and Friends Management Care Centre (EFMC), a NGO based in Abuja, Nigeria, conducted health talks, free malaria testing, distribution of insecticide-treated mosquito nets and provided free drugs to women and children at three primary health care centers (PHC), in and around the Abuja Nigerian Federal Capital Territory. This report is a description of the events at these three PHCs.

## Methods

**Figure 1:**
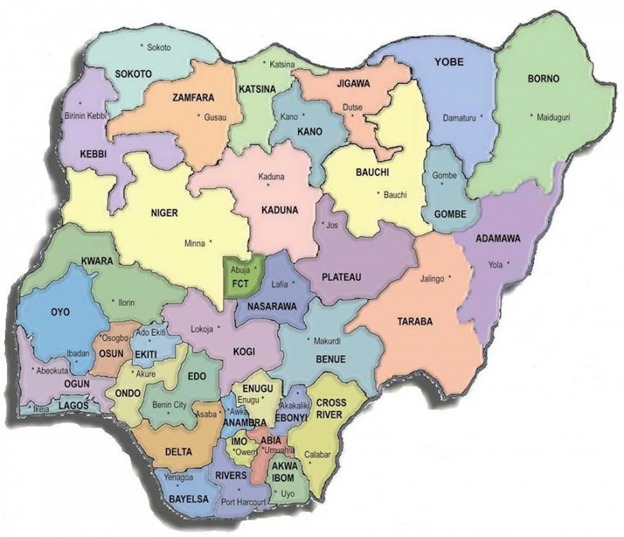
Map of Nigeria showing the Federal Capital Territory (FCT), Abuja where the project was implemented

**Figure 2:**
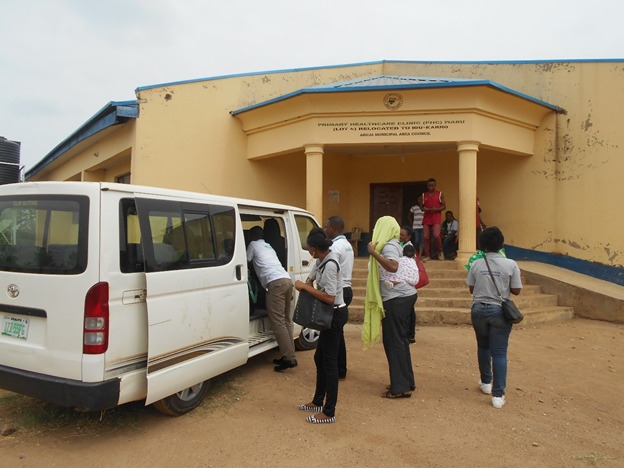
Arrival of EFMC/CFHI team at Primary Healthcare Clinic (PHC) Idu-Karmo, Abuja Nigeria

Excellence and Friends Management Care Centre (EFMC) partnered with the Center for Family Health Initiative (CFHI) in Abuja in the implementation of free medical services to pregnant and nursing women, and children under the age of 5 yrs living in and around the Nigerian capital city. Mosquito nets and rapid diagnostic kits were provide by EFMC, CFHI and the Nigerian National Malaria Elimination Program (NMEP). Following several preparatory meetings, three sites-Kagini, Gwagwa and Idu-Karmo Primary Health Care Centers were purposefully selected (Figure 1). Criteria for selection included location, population covered and antenatal/well baby clinics on World Malaria Day (WMD). Selected sites were informed of their choice as well as the plans to provide free medical services with ITN distribution on WMD. Following consent, EFMC and CFHI designated staff members were divided into three teams led by Program Managers or clinical associates. Each team of 10 persons had testers, counsellors, doctors, community health workers and other support staff. Activity at each center was to start as early as 0800 hours and continued until the antenatal or well-baby clinic was over. EFMC/CFHI staff worked with staff of the facility to ensure their full involvement and proper documentation of the exercise Figure 2.

## Results

A total of 117 women (pregnant women and nursing mothers) were enrolled with free medical services at the three centers-Kagini (40), Gwagwa (42) and Idu-Karmo (35) Primary Health Care Centers Table 1. Health prevention talks, malaria diagnosis, provision of malaria medications and distribution of mosquito nets were ear marked for each center.

**Table 1:** Women reached, tested for malaria with RDT and given ITN

**Facility**	**No of People Reached**	**No Tested**	**No that Received ACT**	**No that Received ITNs**
Kagini	40	32	Nil	20
Idu-Karmo	35	20	8	33
Gwagwa	42	27	Nil	25
Total	117	79	8	78

**Figure 3:**
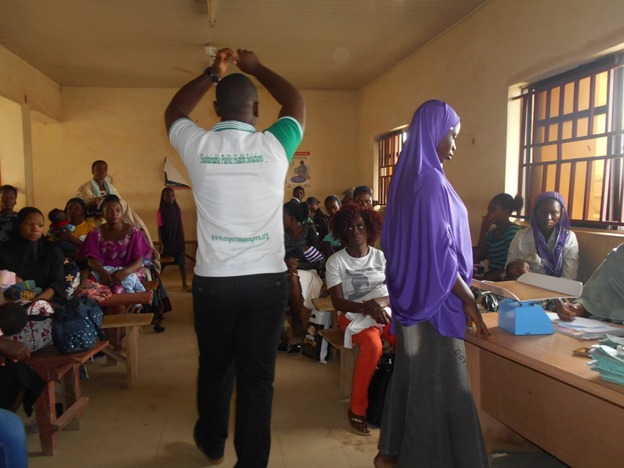
Health talk on malaria and better health-seeking behavior, PHC Idu-Karmo

**Health talks**: The program began with a health talk upon the arrival of the project team at the designated facility. In most facilities, the beneficiaries, made up of women and children, were already seated in anticipation of the proposed medical services. Health talks on healthy living, malaria symptoms, preventive strategies including use of ITNs, indoor residual sprays and malaria chemoprophylaxis for the vulnerable population were undertaken; and management of fevers were presented to the participants by qualified healthcare workers. Strategies to prevent malaria infection by reducing mosquito bites were highlighted including through the use of mosquito nets. Attendees were encouraged to prevent malaria by embracing the use of treated mosquito nets and maintaining them appropriately. Participants were also advised to maintain clean environments devoid of breeding grounds for mosquitoes. Several questions on how to effectively use mosquito nets where posed by attendees and responded to by the project management team Figure 3.

**Figure 4:**
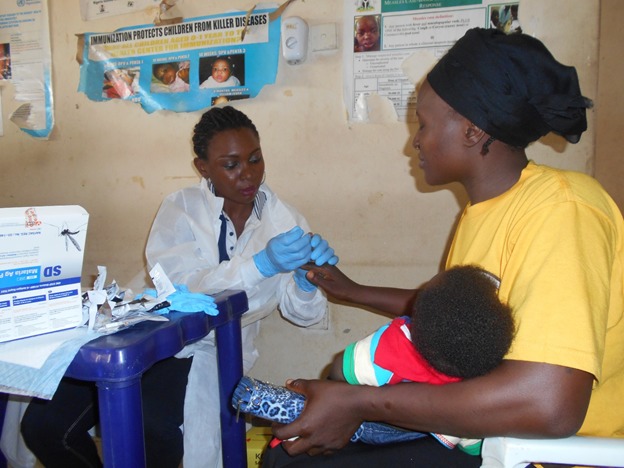
Malaria rapid diagnostic test at PHC Idu Karimo

**Figure 5:**
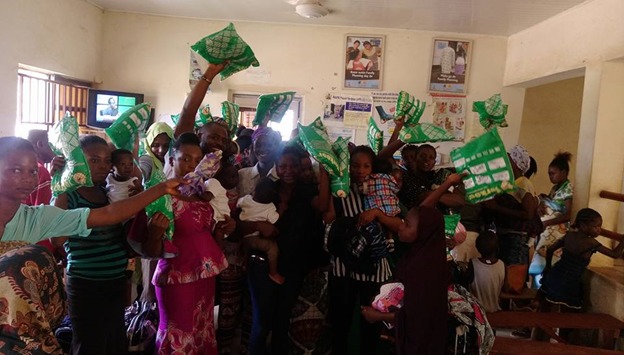
Mothers displaying their ITNs at PHC Gwagwa, Abuja

**Malaria diagnosis and ITN distribution**: Free malaria tests were conducted using rapid test kits (One Step Malaria P.f HRH -II Antigen Rapid TEST One Step Malaria P.f HRH-II Antigen Rapid TEST [2] for 79 (67.5%) women and children who had a febrile illness. Similarly, 78 (66.7%) insecticide-treated mosquito nets were distributed to qualified pregnant women and nursing mothers Figure 4, Figure 5.

**Outcome of malaria testing**: None of the women and children tested was identified to have malaria from the test. This is despite the fact that they had clinical symptoms similar to malaria like fever.

**Responses from beneficiaries**: Beneficiaries were excited about the services and knowledge acquired on how to prevent malaria. The beneficiaries also took turns to show their appreciation in their local languages from several ethnic groups of Nigeria. In the words of some of the beneficiaries: “We are glad for the knowledge acquired today, the free test and free net. Thank God someone remembered us in recession” LE “What happened today is a good thing. I have leant how I can protect my family” MU “We give God thanks for the good works of EFMC” RA.

## Discussion

World Malaria Days are dedicated days designed to help improve awareness of the impact of malaria, prevention strategies and treatment options available in the communities. Our activity showed that not all malaria-like fevers in malaria-endemic regions like Nigeria are as a result of malaria [3, 4]. There are several publications on the dangers of presumptive or clinical diagnosis of malaria. There are several publications on the dangers of presumptive or clinical diagnosis of malaria, resulting in false positive diagnosis and unnecessary treatment. In a study in Lagos, Nigeria, over-diagnosis and over-treatment of malaria was seen, of children treated with antimalarials were confirmed to be parasite positive as only 251 of 853 (29.4%) of children treated with antimalarials were confirmed to be parasite positive [3]. In a similar study on malaria burden in the neighbouring Republic of Niger, the number of cases of presumed malaria reported in health centers were said to be largely overestimated as a result of inadequacies in the clinical case ascertainment of the malaria and of disease risk, as patients with simple febrile illness were often wrongly classified as malaria cases [4]. In our own field work, although the subjects had clinical pyrexia, malaria tests were negative, again confirming that many fevers in Nigeria may not be as a result of malaria. Studies from Tanzania [5], Namibia [6] and several other nations have documented similar findings which all shown that diagnosis of malaria using only clinical means leads to over-diagnosis [7]. In the Tanzanian study, malaria was the clinical diagnosis for 528 (60.7%), but was the actual cause of fever in only 14 (1.6%) [5]. Whereas in Namibia, only 36% of patients admitted for malaria were confirmed as malaria [6]. Beyond exposure to the adverse effects of an unwarranted antimalarial, a prospective study in Tanzania documented higher case fatality rate for slide negative, than slide positive people (p < 0.001) [8]. Reyburn and colleagues were of the view that malaria is commonly over-diagnosed in people presenting with severe febrile illness, especially in those living in areas with low to moderate transmission and in adults resulting in failure to treat alternative causes of severe infection. This failure commonly results in avoidable deaths.

As clinical diagnosis has very low sensitivity and specificity due to the nonspecific nature of the symptoms and signs of malaria, this informed the development of the current malaria treatment guidelines that emphasized parasitic diagnosis rather than clinical diagnoses [9-12]. There is therefore, the need, in line with the current malaria treatment guidelines of Nigeria [9, 10], Sierra Leone [11], Liberia [12] and several other countries in malaria-endemic areas to ensure parasitic diagnosis before treatment for malaria is commenced as parasite-based diagnosis is the gold standard. Beyond diagnosis, quantification and speciation of parasites allow for improved patient care in malaria-positive patients, identification of malaria-negative patients in whom another diagnosis must be explored, avoidance of unnecessary antimalarial intake with resultant reduction in adverse effects, drug interactions and selection pressure for resistance. In addition, this will result in improved health information and proper confirmation of treatment failures. Use of RDT has its advantages which include the fact that it does not require any equipment or expertise as community health workers and field staff used it successfully, results are available within 15 to 20 minutes, can be done at the consulting room/bedside and can be used in settings where microscopy is not available. Variation in sensitivity and specificity of the various RDTs, with high levels of false negative results in those with low parasite densities and or due to prozone effect is a major drawback. Also, false positive results due to recent infection as antigen clears slowly as well as from heterophile antibodies limits the usefulness of this process. In addition, poor storage of the RDT can increase the rate of false positive or negative results as there is the need to keep the RDT cassettes/strips at low temperature. The limitations of RDT can be minimized by the use of microscopy diagnosis which is more cost effective in high transmission areas as it is cheaper, allows quantification and speciation of parasites, allows for detection of treatment failures, and allows for detection of other causes of fever and other diseases found in the area. However, microscopic diagnosis is limited by the following challenges-needs functional microscope and power source and expertise at reading stained blood smears. It is also tedious and time consuming resulting in delayed results that may also be due to high load of fever cases in the community.

## Conclusion

The impact of the knowledge shared and rapid test carried out was evident during the field work. As severe malaria is clinically similar to other severe febrile illnesses and in endemic areas, parasitological confirmation of parasitaemia is often unavailable or unreliable, false-positive malaria microscopy is therefore very common. This most time results in wrong focus on malaria with resultant unnecessary antimalaria treatment and failure to address other life-threatening conditions [13]. Over diagnosis has been linked to the influence of initial training within a context where the importance of malaria is strongly promoted; the influence of peers, conforming to perceived expectations from colleagues; pressure to conform with perceived patient preferences; and quality of diagnostic support, involving resource management, motivation and supervision [14]. Following the dictates of the guidelines-rather than “mindlines” [14] should be encouraged across all levels of care. Finally, to sustain the gains of the World Malaria Day, measures should be taken to broaden the reach to ensure sustained asses to information by the general public to prevent malaria infection in our communities, as well as parasitic diagnosis to minimize over diagnosis of malaria in infants, pregnant and nursing mothers.

## Competing interests

The authors declare no competing interests.
